# Efficient Navigable Area Computation for Underground Autonomous Vehicles via Ground Feature and Boundary Processing

**DOI:** 10.3390/s25175355

**Published:** 2025-08-29

**Authors:** Miao Yu, Yibo Du, Xi Zhang, Ziyan Ma, Zhifeng Wang

**Affiliations:** 1 School of Mechanical and Electrical Engineering, China University of Mining and Technology-Beijing, Beijing 100083, China; bqt2000401011@student.cumtb.edu.cn; 2 Coal Mining Research Institute, China Coal Technology Engineering Group, Beijing 100013, China; duyibo@tdkcsj.com (Y.D.); maziyan@tdkcsj.com (Z.M.); kdbjwzf@tdkcsj.com (Z.W.)

**Keywords:** autonomous driving, underground mines, 3D point cloud, boundary detection, direction extraction

## Abstract

Accurate boundary detection is critical for autonomous trackless rubber-wheeled vehicles in underground coal mines, as it prevents lateral collisions with tunnel walls. Unlike open-road environments, underground tunnels suffer from poor illumination, water mist, and dust, which degrade visual imaging. To address these challenges, this paper proposes a navigable area computation for underground autonomous vehicles via ground feature and boundary processing, consisting of three core steps. First, a real-time point cloud correction process via pre-correction and dynamic update aligns ground point clouds with the LiDAR coordinate system to ensure parallelism. Second, corrected point clouds are projected onto a 2D grid map using a grid-based method, effectively mitigating the impact of ground unevenness on boundary extraction; third, an adaptive boundary completion method is designed to resolve boundary discontinuities in junctions and shunting chambers. Additionally, the method emphasizes continuous extraction of boundaries over extended periods by integrating temporal context, ensuring the continuity of boundary detection during vehicle operation. Experiments on real underground vehicle data validate that the method achieves accurate detection and consistent tracking of dual-sided boundaries across straight tunnels, curves, intersections, and shunting chambers, meeting the requirements of underground autonomous driving. This work provides a rule-based, real-time solution feasible under limited computing power, offering critical safety redundancy when deep learning methods fail in harsh underground environments.

## 1. Introduction

With the growing global demand for coal, mining activities are increasingly shifting from open-pit to underground operations. However, underground mining faces inherent challenges, including lower operational efficiency and heightened safety risks due to the complex subsurface environment. The adoption of autonomous transportation systems in underground mines offers a transformative solution: it not only enhances operational efficiency but also minimizes human involvement, thereby significantly improving worker safety [1]. Consequently, advancing autonomous transportation technologies tailored for underground mining environments has become a critical research priority with substantial practical and academic value.

Tunnel boundary detection stands as a cornerstone technology for autonomous underground transportation. By accurately identifying tunnel boundaries, autonomous vehicles can maintain precise positioning [2] relative to the tunnel structure, thereby preventing catastrophic lateral collisions with tunnel walls. Existing boundary detection methods are broadly categorized into image-based and LiDAR-based approaches [3]. Image-based methods typically leverage deep convolutional neural networks trained on manually annotated datasets to perform semantic segmentation of road regions [4,5,6,7]. While images provide rich semantic details and high-resolution visual information, their reliability degrades severely in underground environments due to poor illumination, water mist, and airborne dust—factors that introduce noise and obscure critical features, undermining boundary detection accuracy [8]. Furthermore, 2D images inherently lack depth information, making it challenging to quantify the precise distance between the vehicle and tunnel boundaries, a limitation that compromises collision avoidance efficacy.

In contrast, LiDAR systems, equipped with active light sources, are impervious to ambient lighting conditions. Their laser beams can penetrate water mist and dust particles, enabling the acquisition of high-fidelity 3D point clouds even in harsh underground environments [9,10]. Previous studies have explored LiDAR-based boundary detection. Li et al. proposed a structured road boundary detection algorithm that generates super-voxels from point cloud attributes and extracts 3D boundaries using α-shape and energy minimization techniques [11]. Mi et al. developed a voxel-based method to identify candidate curbs, which are then clustered, classified, and fitted for boundary tracking—demonstrating efficacy on sloped roads but failing to account for ground unevenness, a prevalent issue in underground settings [12]. Yu et al. addressed particle degeneration in sparse point cloud scenarios by introducing lateral particle shifting in particle filtering, though their validation was limited to low-traffic urban expressways [13]. For unstructured environments like open-pit mines, Lu et al. proposed a real-time method that extracts boundary points from elevated ground point clouds using spatial and angular features [14]. However, these approaches are primarily designed for open or structured environments and overlook the unique complexities of underground tunnels, such as irregular terrain, dynamic width variations, and discontinuous boundaries in junctions and shunting chambers.

Underground tunnels pose a constellation of distinct challenges that render conventional boundary detection methods inadequate, necessitating the development of specialized technical solutions. Foremost among these is the profound impact of topographic irregularities: uneven surfaces and abrupt slopes introduce significant distortions in point cloud data, complicating the accurate extraction of bilateral boundaries by disrupting the geometric continuity critical for reliable edge detection. Compounding this issue is the dynamic variability of tunnel morphology—frequent width fluctuations, coupled with the presence of chambers and occluded regions, often lead to partial or complete loss of boundary features in point cloud data, creating discontinuities that traditional extraction algorithms struggle to resolve. Further exacerbating these challenges is the need for robust performance under dynamic operational conditions, where vehicle motion artifacts and environmental disturbances introduce temporal instability, demanding not just one-time detection but persistent, high-fidelity tracking of boundary features to ensure continuous safety.

The main contributions of this paper are as follows:

1. A rapid ground orientation extraction method is developed to enable efficient initialization of spatial relationships between LiDAR data and tunnel terrain, laying a foundational coordinate reference for subsequent processing;
2. A real-time coordinate correction framework is proposed, which achieves alignment between the LiDAR coordinate system and the ground plane through a two-stage mechanism of pre-calibration and dynamic feedback adjustment;
3. An adaptive boundary completion algorithm is designed to address boundary discontinuities in complex scenarios, ensuring topological integrity of extracted boundaries;
4. A method for continuously extracting boundaries over duration is proposed. By fusing temporal context information, the continuity of boundary detection is maintained in a dynamic environment.

## 2. Related Work

### 2.1. Image-Based Road Boundary Detection

Image-based algorithms typically rely on edge detection operators or deep neural network models to identify road boundaries. Maddiralla et al. proposed a road detection method based on a convolutional attention mechanism, designed specifically for curved roads, roads with broken lanes, or roads without lanes [15]. This method consists of three main components: an encoder, an enhanced convolutional attention module, and a decoder. The encoder extracts features from the input image, while the enhanced convolutional attention module refines these features for improved quality. Finally, the decoder generates the road detection results, accurately identifying road boundaries in complex scenarios. Swain et al. developed a lane detection approach utilizing the YOLOv5 segmentation model with a ResNet50 backbone network [16]. ResNet50 effectively extracts high-level features from images, while the YOLOv5 model enhances nonlinearity and refines spatial information for better accuracy. Furthermore, the method incorporates data augmentation during preprocessing to improve the model’s robustness and adaptability. Zou et al. proposed a hybrid deep learning architecture for lane detection by leveraging consecutive multi-frame images [17]. The method combines convolutional neural networks (CNNs) and recurrent neural networks (RNNs), where the CNN extracts features from individual image frames, and the RNN processes these features sequentially to establish temporal associations and predict lane positions with improved accuracy. Huang et al. proposed an algorithm for lane extraction on unstructured straight and curved roads [18]. The process begins by converting RGB images into grayscale, followed by smoothing the image using Gaussian and mean filters. For straight lanes, edge detection combined with the Hough Transform is employed, while curved lanes are extracted using an enhanced region growing method and the least squares technique, ensuring accurate lane detection in diverse scenarios.

Although images can provide high-resolution environmental information, the low lighting conditions, water mist, and dust in underground tunnels significantly hinder the performance of image-based road boundary detection algorithms, making it challenging to achieve reliable results.

### 2.2. LiDAR-Based Road Boundary Detection

LiDAR is a high-precision active sensor that operates independently of light and shadows, making it ideal for enabling autonomous driving in complex environments. LiDAR-based road boundary detection methods can be broadly divided into scan-line-based methods and region-of-interest-based methods. The first method involves analyzing the point cloud of each layer in the horizontal direction, identifying road boundary points based on geometric features, and generating the drivable area. The second method extracts regions of interest from the global point cloud and detects the road boundaries.

Han et al. [19] put forward an algorithm for road boundary detection using a single-line LiDAR mounted diagonally downward. It should be noted that single-line LiDAR, due to its structural characteristics, can only capture a single layer of point cloud data, which leads to a lack of height constraints and limits the acquisition of elevation information. This algorithm starts with feature extraction and classification of point cloud data to identify points that belong to the road boundary. Subsequently, the detected road boundaries are tracked using a joint probabilistic data association filter, with vehicle motion information incorporated to adjust detection performance. Wijesoma et al. [20] employed a single-line LiDAR combined with an extended Kalman filter algorithm for road edge detection and tracking. Similarly, the single-line LiDAR here, being constrained by its single-layer detection capability, suffers from insufficient height constraints and cannot provide adequate elevation information, and the integration of the filter forms a notable aspect of their approach.

Liu et al. [21] proposed a method for detecting road drivable areas by fusing images and point clouds. The specific process is as follows: first, the image is divided into superpixels, and the point cloud is projected onto the image. Within each superpixel block, the point cloud is triangulated, and the average of the normal vectors of all triangles adjacent to a single LiDAR point is computed as the normal vector of that point. Then, the angle between this normal vector and the ground is used to determine whether the point is an obstacle. Finally, the drivable area is generated by starting from the pixels in the middle and bottom of the image and extending outward to both sides; the extension stops when an obstacle boundary is encountered, thus obtaining the final drivable area. Rato et al. [22] defined a region of interest with a certain range in front of the vehicle and to its sides. They then converted the point cloud within this region into a 2D grid map and adopted an image-based method to detect road boundaries.

## 3. Methods

**Figure 1:**
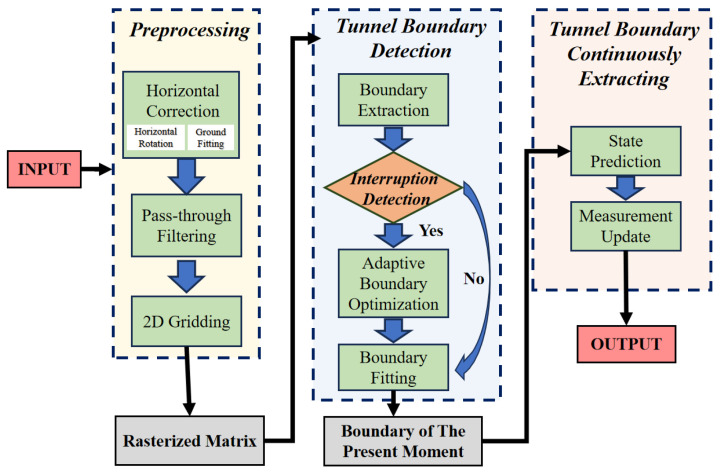
Overall workflow for underground tunnel boundary detection.

To address the unique challenges of underground tunnel boundary detection—including topographic irregularities, boundary discontinuities, and dynamic tracking requirements—this paper proposes a LiDAR-based detection and tracking framework that integrates spatial correction, grid-based projection, and temporal context fusion. Figure 1 illustrates the core workflow of the proposed underground tunnel boundary detection method, which consists of three interconnected modules: preprocessing, tunnel boundary fitting, and boundary continuously extracting. This step isolates ground point clouds from non-ground features through a fast segmentation strategy, laying the groundwork for subsequent processing. The extracted ground point clouds then undergo further correction—via pre-calibration and dynamic updates—to align with the LiDAR coordinate system, ensuring spatial parallelism. Following this, the corrected ground point clouds are projected to generate a 2D gridded point cloud, structuring the spatial information to facilitate robust boundary extraction. Subsequently, the gridded point cloud is fed into the boundary extraction module to identify initial tunnel boundaries. A scene analysis step is then incorporated to detect the presence of curves, intersections, or shunting chambers. In cases where such complex structures are absent, the process advances directly to boundary fitting. Conversely, if curves, intersections, or shunting chambers are detected, a compensation mechanism is activated to supplement missing point cloud data in occluded regions prior to boundary fitting. This adaptive strategy ensures the continuity and accuracy of boundary information even in scenarios with partial occlusions, such as curves, intersections, and shunting chambers. Finally, the fitted boundary is subjected to tracking via the Kalman filter algorithm, enhancing the stability of tunnel boundary detection results under dynamic conditions.

### 3.1. Preprocessing Module

The quality of the preprocessing module is critical for ensuring accurate subsequent analysis and processing. To enhance the precision of boundary fitting in the acquired underground tunnel point cloud data, this paper incorporates horizontal correction and 2D gridding as key preprocessing steps.

#### 3.1.1. Horizontal Correction

To capture point cloud data covering a larger perception range, the LiDAR sensor must be mounted at a certain height above the ground with a downward tilt relative to the horizontal plane. However, due to the uneven nature of underground tunnels, this tilt angle continuously changes as the sensor moves. To ensure accurate mapping of the point cloud data onto a 2D grid, this paper introduces a real-time horizontal correction method that adapts to the varying tilt angle. The proposed method comprises three steps: pre-correction, plane model fitting, and plane transformation.

**Figure 2:**
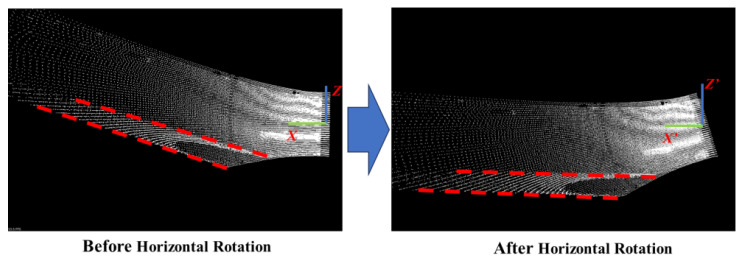
The LiDAR coordinate system XYZ of point clouds is horizontally rotated into the horizontal rotation coordinate system $X^{\prime }Y^{\prime }Z^{\prime }$.

The coordinate systems mainly involve the definition of the LiDAR coordinate system (XYZ), horizontal rotation coordinate system ($X^{\prime }Y^{\prime }Z^{\prime }$), vehicle coordinate system $\left(X_{V}Y_{V}Z_{V}\right)$, and ground coordinate system $\left(X_{G}Y_{G}Z_{G}\right)$, as shown in Figure 2. The ground coordinate system takes the local ground map as the origin, with the vehicle’s forward direction as the $X_{G}$-axis and the left side as the $Y_{G}$-axis. The vehicle coordinate system takes the center of the rear axle as the origin, with the vehicle’s forward direction as the $X_{V}$-axis and the left side as the $Y_{V}$-axis. The horizontal rotation coordinate system is a coordinate system obtained by rotating the X−Y plane along the *Y*-axis with the origin of the LiDAR coordinate system as the rotation center so that it is parallel to the $X_{V}Y_{V}$ plane of the vehicle coordinate system.

**Figure 3:**
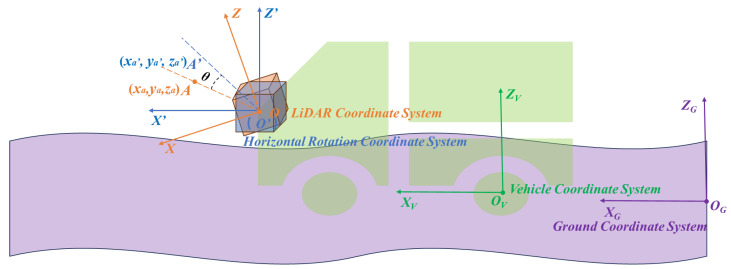
The angle θ between the LiDAR coordinate system XYZ and the horizontal rotation coordinate system $X^{\prime }Y^{\prime }Z^{\prime }$.

Horizontal rotation:

The purpose of horizontal rotation is to initially align the *X*-*Y* plane around the *Y*-*axis* of the LiDAR coordinate system as closely as possible to the horizontal plane of the vehicle coordinate system, as illustrated in Figure 2. This step provides a better starting point for the subsequent iterative optimization of the plane model, reducing the likelihood of convergence to local optima. As illustrated in Figure 3, the coordinates of point cloud A in the LiDAR coordinate system are denoted as $\left(x_{a},y_{a},z_{a}\right)$. The angle θ between the LiDAR and the horizontal plane is determined through manual measurement, while the yaw and roll angles of the LiDAR are disregarded. Using this information, the rotation matrix $R_{1}$ is derived, as shown in Equation (1).

(1) $$R_{1}=\left[\begin{array}{cccc} 1 & 0 & 0 & 0\\ 0 & \cos\theta & \sin\theta & 0\\ 0 & -\sin\theta & \cos\theta & 0\\ 0 & 0 & 0 & 1 \end{array}\right]$$

Subsequently, the coordinates of point cloud A are transformed using the rotation matrix $R_{1}$, as shown in Equation (2). This transformation yields the horizontal rotation coordinates of point cloud A, denoted as $A^{\prime }\left(x_{a}^{\prime },\;y_{a}^{\prime },\;z_{a}^{\prime }\right)$. The transformation process is carried out as follows:

(2) $$\left[\begin{array}{c} x_{a}^{\prime }\\ y_{a}^{\prime }\\ z_{a}^{\prime }\\ 1 \end{array}\right]=\left[\begin{array}{cccc} 1 & 0 & 0 & 0\\ 0 & \cos\theta & \sin\theta & 0\\ 0 & -\sin\theta & \cos\theta & 0\\ 0 & 0 & 0 & 1 \end{array}\right]\left[\begin{array}{c} x_{a}\\ y_{a}\\ z_{a}\\ 1 \end{array}\right]$$

Rapid Ground Model Fitting via Adaptive Planar Segment Analysis:

Before conducting plane model fitting, an area-based filtering algorithm is applied to reduce the volume of point cloud data and improve runtime efficiency. The filtered point cloud, representing the current ground points, is denoted as set *Q*.

The extraction of the ground model establishes a spatial reference computation of the navigable area in underground tunnels. This framework extracts ground-oriented planar features from LiDAR point clouds, optimized for limited computing power, integrating adaptive neighborhood search, geometric consistency validation, and efficient normal vector propagation.

Local surface patches are first extracted by dynamically adjusting the neighborhood search range for each target point *P* (with distance D(P) from the sensor) via a logarithmic model: R(P)=a·log(b·D(P)+1).

Here, *a* scales the range to match tunnel width, and *b* adjusts the growth rate to avoid over-expansion for distant points, both calibrated via offline tests. A valid patch includes the target point plus four cardinal-direction neighbors within $\left[\alpha_{1},\alpha_{2}\right]$ to filter noise, capturing local depth variations.

Planar patches are filtered using horizontal–vertical collinearity checks. For each patch, three key points are evaluated: a central reference point $p_{0}$, and two adjacent points ($p_{1},p_{3}$ horizontally; $p_{2},p_{4}$ vertically). Horizontal consistency uses depth differences and lateral distances from $p_{0}$, with a compensation factor for minor sidewall inclination. Vertical consistency follows a similar logic, with a factor to offset small ground undulations. Patches with both metrics below a threshold are classified as stable planar.

**Figure 4:**
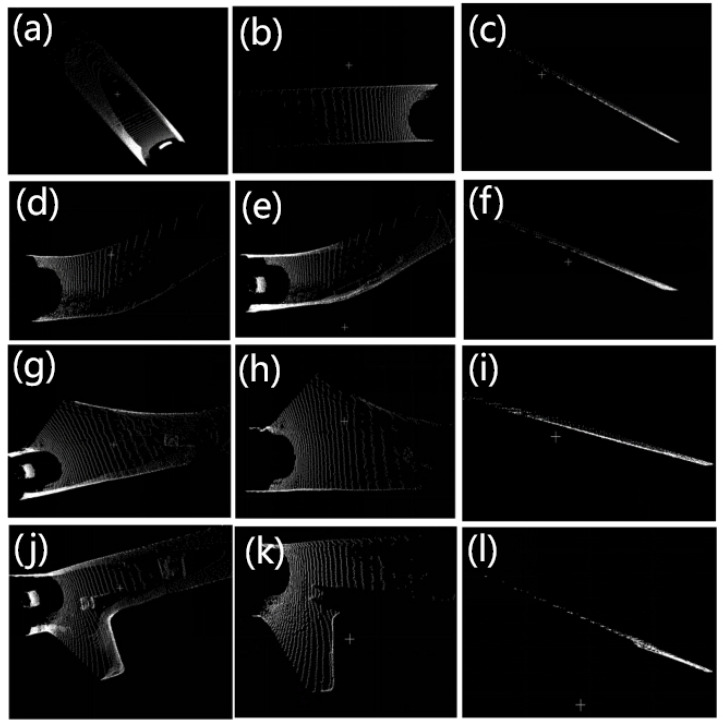
Plane extraction results under four typical scenarios. (**a**,**d**,**g**,**j**) show the original point clouds of straight tunnel, curved tunnel, intersection, and shunting chambers scenarios, respectively. The subsequent two figures in each row represent the top view (**b**,**e**,**h**,**k**) and side view of the extracted ground (**c**,**f**,**i**,**l**).

Normal vectors of these stable patches are derived via covariance matrix eigen decomposition (using the smallest eigenvalue’s eigenvector) and propagated to adjacent patches, reducing redundant calculations. Conflicting norms in overlapping areas trigger re-evaluation of the intersection. This framework prioritizes planar features critical for navigable areas, balancing accuracy and efficiency in resource-constrained underground environments. The results of plane extraction in four typical scenarios are shown in the Figure 4.

Plane transformation: The plane transformation method proposed transforms the original LiDAR point cloud from a horizontal rotation coordinate system ($X^{\prime }Y^{\prime }Z^{\prime }$) into a plane transformation coordinate system ($X^{\prime \prime }Y^{\prime \prime }Z^{\prime \prime }$), which transforms the $X^{\prime }$-$Y^{\prime }$ plane around the $Y^{\prime }$-axis of the horizontal rotation coordinate system as closely as possible to the horizontal plane aligned with the real-time ground plane, achieved by dynamically fitting the actual road surface conditions.

**Figure 5:**
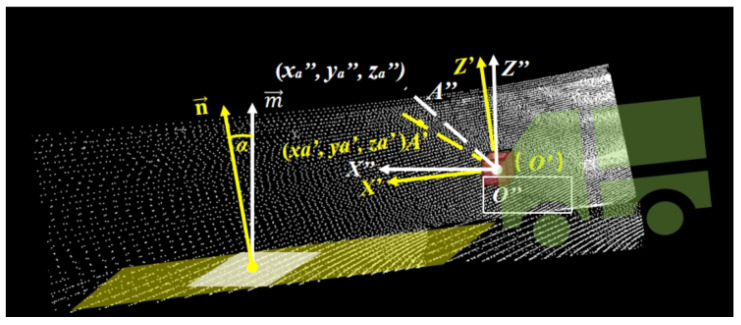
The angle α between the plane normal vector $\vec{n}$ and the normal vector of the real-time ground $\vec{m}$. White indicates the plane transformation coordinate system, while yellow indicates the horizontal rotation coordinate system.

After processing the point cloud set *Q* with the plane model, a plane normal vector $\vec{n}=\left(A,B,C\right)$ is obtained. Vector $\vec{m}=\left(0,0,1\right)$ represents the normal vector of the real-time ground coordinate system. With $\vec{n}$ and $\vec{m}$, the pitch angle α and the rotation axis $\vec{l}$ between the $Y^{\prime \prime }$-axis and the real-time fitted ground can be calculated, as illustrated in Figure 5.

The calculation process is outlined as follows:

(3) $$\alpha =\arccos(\vec{n}\cdot \vec{m})\;$$

(4) $$\vec{l}=\vec{n}\times \vec{m}=\left(B,-A,0\right)$$

According to *Rodrigues’* rotation formula, the rotation matrix $R_{2}$ can be derived as:

(5) $$R_{2}=\left[\begin{array}{ccc} \cos\alpha +B^{2}\left(1-\cos\alpha \right) & -AB(1-\cos\alpha ) & -A\sin\alpha \\ -AB(1-\cos\alpha ) & \cos\alpha +A^{2}\left(1-\cos\alpha \right) & -B\sin\alpha \\ A\sin\alpha & B\sin\alpha & \cos\alpha \end{array}\right]\;$$

The coordinates of the horizontal rotated point cloud $A^{\prime }\left(x_{a}^{\prime },\;y_{a}^{\prime },\;z_{a}^{\prime }\right)$ in the horizontal rotation coordinate system $X^{\prime }Y^{\prime }Z^{\prime }$ are then transformed using the rotation matrix $R_{2}$. The transformation process is shown in Equation (6), resulting in the s plane transformed point cloud coordinates $A^{\prime \prime }\left(x_{a}^{\prime \prime },\;y_{a}^{\prime \prime },\;z_{a}^{\prime \prime }\right)$ in the plane transformation coordinate system $X^{\prime \prime }Y^{\prime \prime }Z^{\prime \prime }$:

(6) $$\left[\begin{array}{c} x_{a}^{\prime \prime }\\ y_{a}^{\prime \prime }\\ z_{a}^{\prime \prime } \end{array}\right]=R_{2}\left[\begin{array}{c} x_{a}^{\prime }\\ y_{a}^{\prime }\\ z_{a}^{\prime } \end{array}\right]$$

The advantage of this approach is that it reliably transforms 3D point cloud data into a coordinate system consistent with the actual ground plane, even when the vehicle is driving on uneven surfaces, experiencing changes in posture, or encountering significant slopes.

#### 3.1.2. Two-Dimensional Gridding

**Figure 6:**
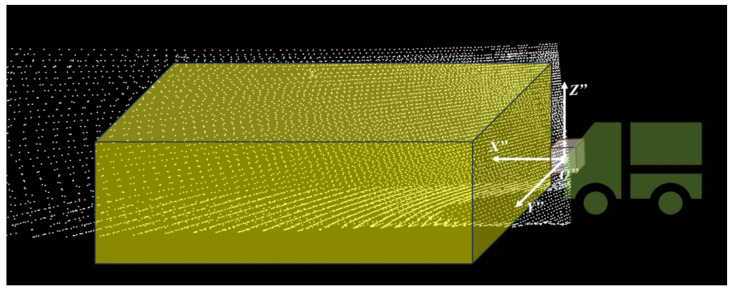
Three-dimensional limits of the point cloud of interest in the transformed plane coordinate system.

To reduce computational complexity and enhance overall efficiency, this paper employs 2D gridding to process the corrected point cloud, as illustrated in Figure 6.

Since this paper focuses on boundary detection, a pass-through filter is applied prior to the gridding process to eliminate points outside the critical area, further reducing computational load. The specific operation of the pass-through filter is as follows: First, a filter object is created, and the filter fields and ranges, denoted as E, are defined. The filter then checks whether the corrected point cloud $A^{\prime \prime }\left(x_{a}^{\prime \prime },\;y_{a}^{\prime \prime },\;z_{a}^{\prime \prime }\right)$ falls within the range *E*, as shown in Equation (7):

(7) $$\left\{\begin{array}{c} x^{\prime \prime }\in \left[x_{min}^{\prime \prime },\;x_{max}^{\prime \prime }\right]\\ y^{\prime \prime }\in \left[y_{min}^{\prime \prime },\;y_{max}^{\prime \prime }\right]\\ z^{\prime \prime }\in \left[z_{min}^{\prime \prime },\;z_{max}^{\prime \prime }\right] \end{array}\right.$$

$x_{min}^{\prime \prime }$ and $x_{max}^{\prime \prime }$ represent the minimum and maximum thresholds of *E* along the $X^{\prime \prime }$-axis in the LiDAR coordinate system; $y_{min}$ and $y_{max}$ are the corresponding thresholds along the Y-axis; and $z_{min}^{\prime \prime }$ and $z_{max}^{\prime \prime }$ are the thresholds along the $Z^{\prime \prime }$-axis. Finally, a filtering operation is performed to retain the points within the specified range, resulting in the point cloud set $\{p_{i}\left(x_{i}^{\prime \prime },\;y_{i}^{\prime \prime },\;z_{i}^{\prime \prime }\right)\}\subset E$.

For the boundary extraction task, consideration must be given to positions on the horizontal plane. Thus, data along the $Y^{\prime \prime }$-axis is retained up to the maximum width of general tunnel designs, with pass-through filtering applied exclusively to the $X^{\prime \prime }$-axis and $Z^{\prime \prime }$-axis. For the $X^{\prime \prime }$-value (distance), focus is placed on LiDAR point cloud data within the first 0-20 m in front of the vehicle. For the $Z^{\prime \prime }$-value (height), account is taken of the vehicle height (2.3 m) and the LiDAR’s installation position (1.3 m above ground). Using the LiDAR coordinate system as the origin, attention is restricted to point cloud data corresponding to tunnel boundaries within the vehicle’s height range. To ensure safety, additional buffer zones are introduced in the height range: a 0.7 m safety margin is added for uneven ground or downward slopes, while a 0.5 m safety margin is applied for upward slopes or to account for vehicle vibrations.

In practical underground roadway environments, vehicles and pedestrians may interfere with boundary detection results. Therefore, based on actual measurements and point cloud data analysis, the experiment adopted an appropriate filtering range. Specific settings are provided in Table 1.

**Figure 7:**
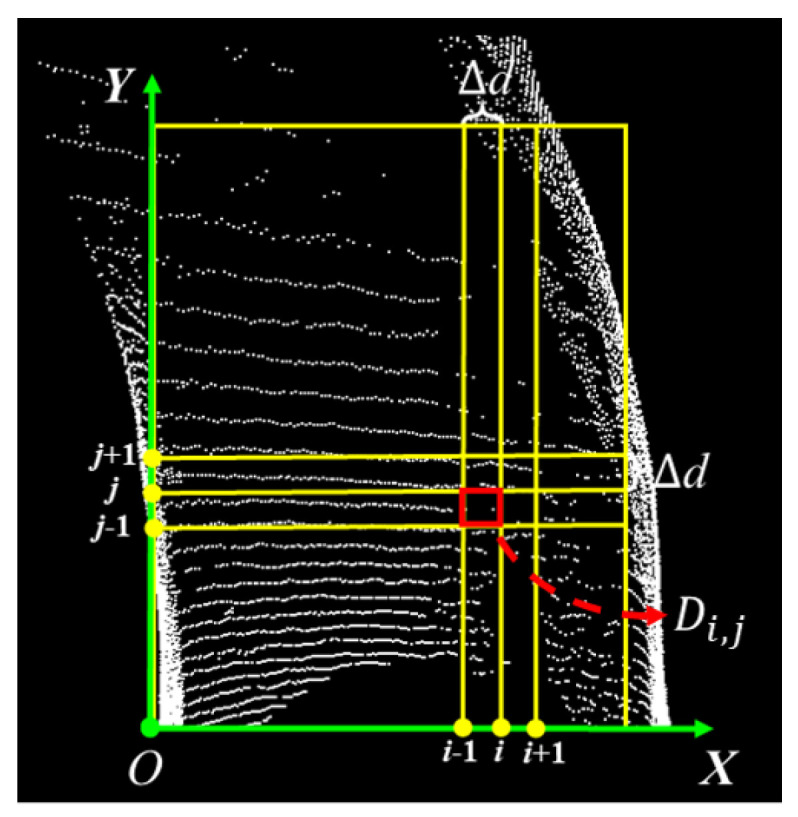
Schematic diagram of the grid map.

After obtaining the point cloud $p_{i}\left(x_{i}^{\prime \prime },y_{i}^{\prime \prime },z_{i}^{\prime \prime }\right)$ that has undergone pass-through filtering, the process of rasterization is initiated. The main steps are as follows: First, a grid map $Map_{max\_{\Delta x}^{\prime \prime },max\_{\Delta y}^{\prime \prime }}$ is initialized, where max_Δx,max_Δy are the predefined dimensions of the grid map. Each cell in the grid map is rectangular, with a uniform length and width of Δd, as illustrated in Figure 7.

Next, each point $p_{i}\left(x_{i}^{\prime \prime },y_{i}^{\prime \prime },z_{i}^{\prime \prime }\right)$ in the filtered point cloud is traversed, and its two-dimensional index within the grid map is calculated. The specific calculation process is shown in Equations (8) and (9):

(8) $$x_{id}^{\prime \prime }=\frac{x_{i}^{\prime \prime }-\min\left(x^{\prime \prime }\right)}{\max\left(x^{\prime \prime }\right)-\min\left(x^{\prime \prime }\right)}$$

(9) $$y_{id}^{\prime \prime }=\frac{y_{i}^{\prime \prime }-\min\left(x^{\prime \prime }\right)}{\max\left(x^{\prime \prime }\right)-\min\left(x^{\prime \prime }\right)}\;$$

It should be noted that $x_{id}^{\prime \prime }$ is the mapped position of $x_{i}^{\prime \prime }$ from the 3D point cloud onto the grid map, and $y_{id}^{\prime \prime }$ is the mapped position of $y_{i}^{\prime \prime }$ from the 3D point cloud onto the grid map.

Finally, it is checked whether $x_{id}^{\prime \prime }$ and $y_{id}^{\prime \prime }$ are within the valid range of the grid map, i.e., if $x_{id}^{\prime \prime }\in \left[\min\left(x^{\prime \prime }-\right),\max\left(x^{\prime \prime }\right)\right]$ and $y_{id}^{\prime \prime }\in \left[\min\left(y^{\prime \prime }-\right),\max\left(y^{\prime \prime }\right)\right]$ are both true, the point will be stored in the corresponding cell. When all points in the point cloud $p_{i}\left(x_{i}^{\prime \prime },y_{i}^{\prime \prime },z_{i}^{\prime \prime }\right)$ have been traversed, the 2D rasterization is complete.

The preprocessing module used in this paper improves data quality, reduces data volume, enhances feature extraction, and increases processing efficiency, facilitating subsequent analysis and processing. After normalization, the point cloud data occupies a fixed and finite coordinate range, enhancing computational efficiency and reducing algorithmic complexity, thus improving real-time performance. Furthermore, normalization ensures the algorithm’s insensitivity to variations in tunnel dimensions; regardless of the actual tunnel size, the point cloud data is consistently mapped into the same scale, ensuring the generality and robustness of subsequent boundary detection algorithms.

### 3.2. Tunnel Boundary Detection

After the original point cloud has undergone the preprocessing module, a representative 2D rasterized matrix is obtained. In this section, the fitting operation begins. The tunnel boundary detection module mainly consists of three main steps: boundary extraction, adaptive boundary optimization and completion, and boundary fitting.

#### 3.2.1. Boundary Extraction

The primary goal of boundary extraction is to extract key points that form the region boundaries from the rasterized data for subsequent precise fitting. This process is divided into two parts: candidate boundary point extraction and outlier removal. Candidate boundary point extraction is undertaken to speed up the subsequent boundary fitting. By rasterizing the point cloud data $p_{i}\left(x_{i},y_{i},z_{i}\right)$, we obtain the grid map $Map_{max\_x^{\prime \prime },max\_y^{\prime \prime }}$, where each cell contains several points, and the left boundary point cloud falls within the column with the smallest non-empty cell index in each row, while the right boundary point cloud falls within the column with the largest non-empty cell index in each row. According to this pattern, the following extraction procedure is adopted:

1. Initialize the left and right boundary point clouds as L and R.
2. Traverse each row $Map_{*,max\_y^{\prime \prime }}$ of $Map_{max\_x^{\prime \prime },max\_y^{\prime \prime }}$, and initialize the minimum and maximum X coordinates $x^{\prime \prime }\_min$, $x^{\prime \prime }\_max$ and their indices.
3. Traverse each column within the row to find the points corresponding to the minimum and maximum X coordinates in the non-empty cells. After finding the points, compare them with $x_{min}^{\prime \prime }$ and $x_{max}^{\prime \prime }$, then update $x_{min}^{\prime \prime }$, $x_{max}^{\prime \prime }$ and their indices accordingly.
4. Store the leftmost point into the left boundary point cloud L and the rightmost point into the right boundary point cloud R.
5. Finally, after traversing all rows, the left boundary point cloud set $L=\{l_{1},l_{2},l_{3},\ldots ,l_{w}\}$ and the right boundary point cloud set $R=\{r_{1},r_{2},r_{3},\ldots ,r_{v}\}$ can be obtained, where $l_{1}$, $l_{2}$, $l_{3}$,…, $l_{w}$ are the 1st, 2nd, 3rd,..., wth clusters of the left boundary, and $r_{1}$, $r_{2}$, $r_{3}$,…, $r_{v}$ are the 1st, 2nd, 3rd,..., vth clusters of the right boundary.

Outlier removal: The main purpose of this step is to further improve the quality of the candidate boundary points and reduce the computational complexity for the subsequent fitting process. In this paper, outliers include outliers and aberrant points in the point cloud. The specific process is as follows (using the left boundary as an example):

1. For each point P∈L, where *L* is the left boundary point cloud set, find the *K* nearest neighbor points of *P*.
2. Calculate the average distance μ and standard deviation σ from P to these K points.
3. Determine if the distance from *P* to the *K* points is greater than μ+0.5σ. If not, it is not considered an outlier.
4. Check if the difference between the X coordinate of *P* and the last point in the point cloud L is less than 1. If yes, it is not an aberrant point.
5. Add points *P* that are determined to be neither outliers nor aberrant points to the output point cloud $L_{1}$, which is the left boundary point cloud set after removing outliers. Similarly, obtain the right boundary point cloud set $R_{1}$ after removing outliers.

The point cloud data processed by the boundary extraction module provides high-quality input for further boundary fitting and other subsequent processing, enhancing the efficiency and effectiveness of the point cloud processing system.

#### 3.2.2. Adaptive Boundary Optimization

In underground tunnels, there may be intersections and shunting chambers. When such road conditions occur, the extracted left or right boundary may be interrupted, affecting the quality of subsequent fitting operations. Therefore, this paper adopts an adaptive boundary completion method to optimize the issue of discontinuous boundary points and reduce errors in subsequent fitting or tracking.

After obtaining the critical boundary points, it is determined whether adaptive boundary optimization is needed. The specific process is as follows: first, traverse and compare each point $P_{L1}$ and $P_{R1}$ of the left and right boundary point clouds $L_{1}$ and $R_{1}$ obtained during boundary extraction; then, calculate the lateral differences for each point and set a threshold. If the difference is greater than the threshold, it is recorded. Finally, if the number of points with lateral differences greater than the threshold exceeds five after traversal, it is recognized that boundary completion is needed.

Adaptive boundary optimization is divided into two parts: foreground boundary extraction and boundary adaptive completion.

Foreground boundary extraction: Foreground boundary extraction is based on the already extracted left and right boundary point clouds. Its main role is to downsample these boundary point clouds and remove points within the road boundary threshold, thereby extracting the portion of the road boundary point cloud that needs to be completed. The specific implementation process is as follows:

1. Perform 2D rasterization on the $X^{\prime \prime }$ coordinates of each point in the left boundary point cloud $L_{1}$ and store them in $L_{1}\_index$; then, perform 2D rasterization on the $X^{\prime \prime }$ coordinates of each point in the right boundary point cloud $R_{1}$ and store them in $R_{1}\_index$.
2. Traverse each point $p_{i}=\left(x_{i},y_{i},z_{i}\right)$ in the input point cloud, calculate $x_{id}^{\prime \prime }$ and $y_{id}^{\prime \prime }$ using Equations (8) and (9), where $x_{id}^{\prime \prime }$ is the mapped position of $x_{i}^{\prime \prime }$ from the 3D point cloud onto the grid map, and $y_{id}^{\prime \prime }$ is the mapped position of $y_{i}^{\prime \prime }$ from the 3D point cloud onto the grid map. If the conditions of Equations (10) and (11) are met, add the point to the output point cloud.

(10) $$0<y_{id}^{\prime \prime }<max\_y^{\prime \prime }$$

(11) $$L_{1}\_index\left[y_{id}^{\prime \prime }\right]+3<x_{id}^{\prime \prime }<R_{1}\_index\left[y_{id}^{\prime \prime }\right]-3$$

The final output point cloud is the extracted discontinuous point cloud, with the left and right boundaries marked as $CrossL_{1}$ and $CrossR_{1}$.

Boundary adaptive completion: After obtaining the discontinuous boundary points, the completion process is started to ensure the continuity and accuracy of the boundaries. The main procedure is as follows:

1. Taking the case of a right boundary interruption as an example, select appropriate parameters for boundary fitting on the right boundary $R_{1}$ extracted in Section 3.2.1 (the specific fitting process will be detailed in Section 3.2.3), resulting in a preliminary fitted right boundary.
2. In $CrossR_{1}$, for the ith point, connect the (i − 1)th point with the (i + 1)th point, and shift the point along a direction perpendicular to this line by a distance equal to the width of the roadway.
3. Add the preliminarily fitted right boundary and the shifted $CrossR_{1}$ together to form the final right boundary. If the left boundary is interrupted, the same approach is used for completion.

#### 3.2.3. Boundary Fitting

After acquiring continuous key boundary points, the boundary fitting process is initiated to convert discrete feature points into a continuous geometric representation. To ensure the fitting results support stable and accurate subsequent tracking, the algorithm adopts an iterative optimization strategy that integrates spatial constraints with statistical fitting.

Specifically, the algorithm first determines the effective range of boundary points by calculating the maximum and minimum values of their X and Y coordinates, thereby establishing spatial constraints based on tunnel structural characteristics. It then performs polynomial curve fitting on randomly sampled subsets of key points, evaluates the fitting residuals against a predefined threshold, and dynamically updates the optimal model by retaining the fitting result with the highest number of inliers. This iterative refinement mechanism effectively filters out noise interference, ensuring the final fitted boundary is both continuous and morphologically consistent with the actual tunnel structure.

Through the fitting operation, a discrete set of key boundary points can be transformed into a continuous and smooth curve or line. In the next section, we will make predictions based on the fitted boundaries.

### 3.3. Continuously Extracting Tunnel Boundaries

To maintain the continuity of boundary extraction in dynamic environments, temporal context information is fused to establish spatiotemporal correlations between successive frames. Given the irregular terrain and dynamic factors inherent in underground tunnels, a state estimation mechanism is employed to ensure the consistency of boundary features across time sequences—this mechanism, while leveraging recursive update logic, primarily serves to reinforce the continuity of boundary extraction rather than emphasizing independent tracking functionality. Prior to implementing this mechanism, the state vector *u* and observation vector *z* at the current timestamp are first derived to provide a foundational basis for maintaining temporal consistency of boundary features. The definition of the state vector *u* is shown in Equation (12):

(12) $$u={\left[a_{2},a_{1},a_{0},\Delta a_{2},\Delta a_{1},\Delta a_{0}\right]}^{T}\;$$

where $a_{2}$, $a_{1}$, $a_{0}$ are the parameters of the left boundary fitting curve, and $\Delta a_{2}$, $\Delta a_{1}$, $\Delta a_{0}$ are the rates of change of $a_{2}$, $a_{1}$, $a_{0}$ within a unit time Δt. Since the fitting curve information of each frame’s boundary is obtained, the observation vector *z* is as shown in Equation (13):

(13) $$z={\left[a_{2},a_{1},a_{0}\right]}^{T}\;$$

This state estimation mechanism operates through two interconnected phases: temporal prediction and measurement update, collectively ensuring the continuity of boundary features across successive frames. In the temporal prediction phase, the state of boundary features at time *t* is inferred from the state at t−1, leveraging the temporal correlation of tunnel structures. The prediction model is formulated as:

(14) $$\begin{array}{c} \hat{u}\bar{t}=F\hat{u}t-1\;P_{\bar{t}}=FP_{t-1}F^{T}+Q\; \end{array}$$

where ${\hat{u}}_{\bar{t}}$ denotes the predicted state vector of boundary features at time *t*; *F* represents the transition matrix encoding the spatiotemporal relationship between consecutive frames; ${\hat{u}}_{t-1}$ is the estimated state vector at t−1; $P_{\bar{t}}$ and $P_{t-1}$ are the prior and posterior covariance matrices, respectively; and *Q* accounts for system noise. Given the constrained morphological variations of underground tunnels, a constant-velocity model is adopted to characterize boundary evolution, leading to the transition matrix:

(15) $$F=\left[\begin{array}{cccccc} 1 & 0 & 0 & dt & 0 & 0\\ 0 & 1 & 0 & 0 & dt & 0\\ 0 & 0 & 1 & 0 & 0 & dt\\ 0 & 0 & 0 & 1 & 0 & 0\\ 0 & 0 & 0 & 0 & 1 & 0\\ 0 & 0 & 0 & 0 & 0 & 1 \end{array}\right]$$

where dt is the time interval between consecutive point cloud frames. The measurement update phase refines the predicted state using real-time observations to maintain consistency with actual boundary features. The update process follows:

(16) $$\begin{array}{c} K_{t}=P_{\bar{t}}H^{T}{\left(HP_{\bar{t}}H^{T}+G\right)}^{-1}\\ \hat{u}t=\hat{u}\bar{t}+K_{t}\left(z_{t}-H\hat{u}\bar{t}\right)\\ P_{t}=\left(1-K_{t}H\right)P\bar{t}\; \end{array}$$

Here, $K_{t}$ denotes the weight coefficient balancing prediction and measurement; *H* is the observation matrix projecting the state vector to the measurement space; *G* represents observation noise; and $z_{t}$ is the observed boundary feature vector at time *t*. To ensure dimensional consistency between predicted and observed quantities, *H* is defined as:

(17) $$H=\left[\begin{array}{cccccc} 1 & 0 & 0 & 0 & 0 & 0\\ 0 & 1 & 0 & 0 & 0 & 0\\ 0 & 0 & 1 & 0 & 0 & 0\; \end{array}\right]$$

This iterative prediction-update process continuously adjusts boundary feature states, enhancing the temporal coherence of extracted boundaries and their adaptability to subtle environmental variations.

## 4. Experiments

### 4.1. Experimental Platform Description

The experiments were conducted on a high-performance desktop workstation. This carefully configured platform ensured three critical advantages for underground road scenario analysis: (1) efficient parallel computation through GPU-accelerated tensor operations; (2) real-time processing capabilities for high-resolution sensor data streams; and (3) reproducible experimental conditions with version-controlled dependencies. The combination of hardware specifications and optimized software stack provided sufficient computational headroom for complex tasks while maintaining thermal stability during prolonged training sessions, making it particularly suitable for autonomous driving applications in challenging subterranean environments, as shown in Table 2.

### 4.2. Data Source and Evaluation Metrics

**Figure 8:**
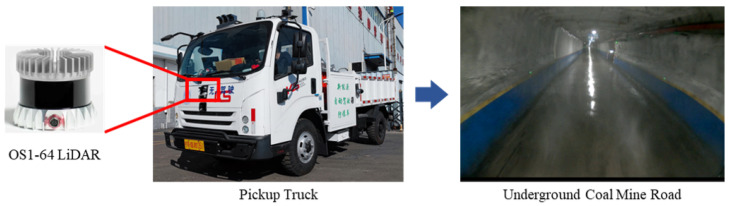
Data collection environment.

The data used in this study were collected from a commonly used transportation road in an underground coal mine production area. The data collection platform was a specialized underground vehicle equipped with an Ouster OS1-64 LiDAR sensor, as shown in Figure 8. Data collection was conducted on a pre-selected, enclosed underground mine road that included four scenarios: straight road, curved road, intersection, and chamber. A total of 8 h of driving data were collected, from which 1200 frames were selected for testing, with 300 frames allocated to each scenario.

**Figure 9:**
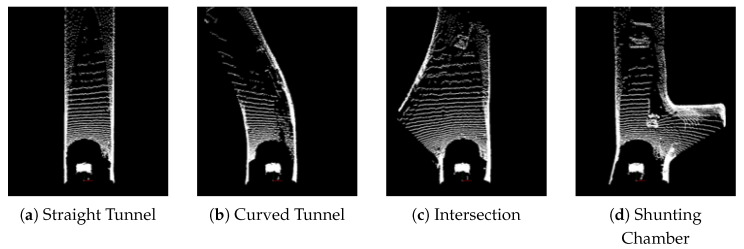
Data scenarios.

The visualization results of the point clouds for the four scenarios are shown in Figure 9. In the straight road scenario, the point cloud appears relatively uniform and continuous. In the curved road scenario, the point cloud distinctly exhibits curved features. For the intersection scenario, the data distribution becomes more complex, displaying multiple branching directions. In the chamber scenario, the point cloud density is significantly higher, reflecting the enclosed and irregular characteristics of the space. These scenarios reveal notable differences in the point clouds, with road boundary detection becoming increasingly challenging as the complexity of the scenarios grows.

To better evaluate the accuracy of underground tunnel boundary fitting, this paper uses manually annotated boundary point clouds as the ground truth. Point clouds within a 2 cm radius of the ground truth are defined as true positive (TP) point clouds, while those outside this range are considered false positive (FP) point clouds. The evaluation metric employed is precision, calculated as shown in Equation (18), where TP represents the total number of true positive point clouds, and FP represents the total number of false positive point clouds:

(18) $$Precision=\frac{TP}{TP+FP}$$

### 4.3. Underground Tunnel Boundary Detection Results

**Figure 10:**
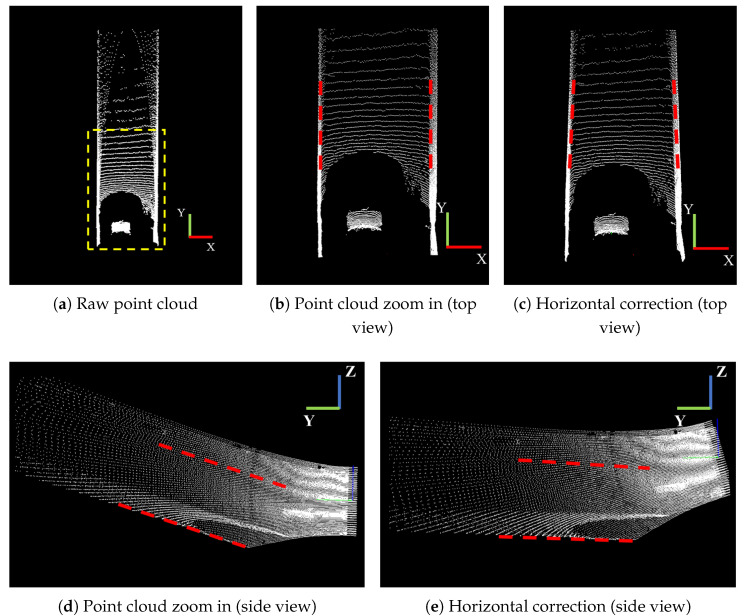
Point cloud horizontal correction result. The area within the yellow dashed line is the calculation zone in front of the vehicle, and the red dashed line represents the reference line for the boundary of the drivable area.

In this subsection, we primarily analyze the boundary fitting results in four scenarios: straight tunnels, curved tunnels, intersections, and shunting chambers. The experimental results for the straight road scenario are illustrated in Figure 10. Figure 10a shows the original point cloud. To clearly demonstrate the subsequent boundary fitting results, the experiment zooms in on the first half of the original point cloud by a factor of two, with the local magnification result presented in Figure 10b. Figure 10c displays the result after horizontal correction. Since it is challenging to observe the effect of the correction from a top view, Figure 10d and Figure 10e, respectively, show the before and after correction results from the side view. After the horizontal correction, the detected boundaries have an inclination angle that is essentially 0 with respect to the horizontal plane, which effectively aids in the subsequent rasterization process and boundary extraction.

**Figure 11:**
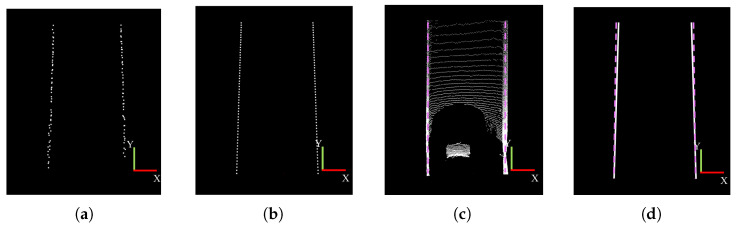
Boundary detection results in a straight tunnel segment. (**a**) Key point extraction; (**b**) boundary fitting; (**c**) ground truth; (**d**) temporal consistency maintenance of boundaries.

Figure 11a shows the extracted key boundary points, which are primarily aligned along a single straight line. Figure 11b,c illustrate the boundary fitting results and the boundary ground truth, respectively. In the straight road scenario, the boundary fitting results closely align with the ground truth, demonstrating high accuracy. Figure 11d presents the boundary tracking results after fitting. To provide a clearer view of the tracking effect, the experiment displays the results at a 2× reduced scale.

**Figure 12:**
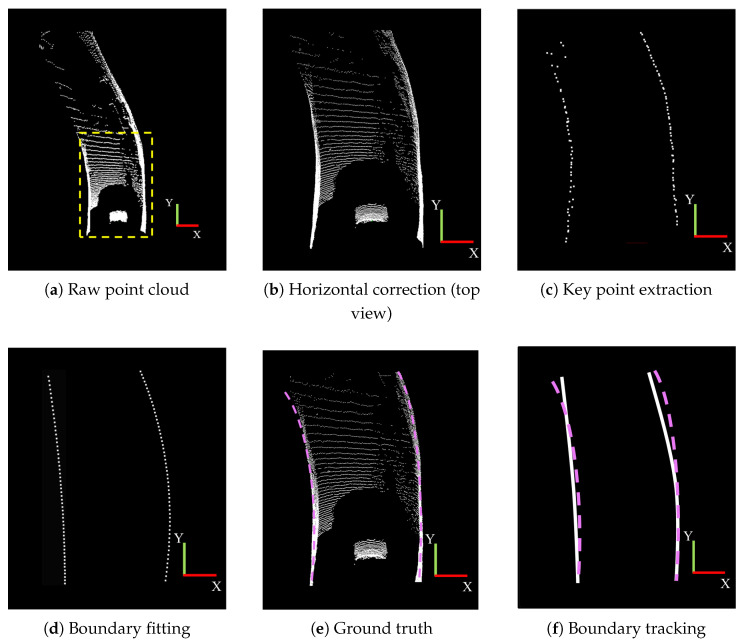
Boundary detection result in the curved road scenario.

The experimental results for the curved road scenario are illustrated in Figure 12. Similar to the straight road scenario, Figure 12a shows the original point cloud, while Figure 12b presents the horizontal correction result of the point cloud in the yellow dotted line area. Figure 12c highlights the key boundary points extracted in the curved tunnel scenario, which collectively form a curve consistent with the characteristics of a curved tunnel. Figure 12d,e depict the boundary fitting results and the ground truth, respectively, demonstrating that the fitting results closely align with the ground truth. Figure 12f illustrates the boundary tracking results, showing that in the curved tunnel scenario, the tracked boundaries accurately follow the actual tunnel boundaries.

**Figure 13:**
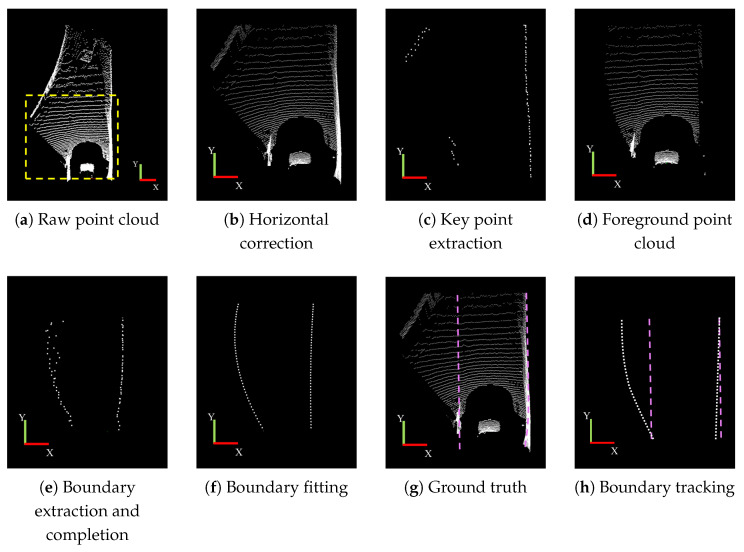
Boundary detection result in the intersection scenario.

The experimental results for the intersection scenario are illustrated in Figure 13. Figure 13a,b display the original point cloud and the result after horizontal correction, respectively. Figure 13c presents the extracted key boundary points in the intersection scenario, revealing a significant interruption on the left boundary. This interruption occurs due to a branch merging into the main path, resulting in a more complex road structure compared to straight and curved tunnels. This complexity makes it challenging to fully extract the boundary points, thereby impacting subsequent boundary fitting.

To address this, the interruption detection step will complete the boundaries in this scenario. Figure 13d shows the foreground of the target point cloud, and Figure 13e presents the boundary completion result, where the completion process ensures continuity of the boundaries on both sides. Figure 13f,g illustrate the boundary detection result and the boundary ground truth, respectively. While the right boundary fitting result closely matches the ground truth, the left boundary fitting shows some deviation due to the intersection on the left side of the road. Figure 13h illustrates the result of boundary tracking, revealing some initial errors in the left-side boundary. However, these errors are quickly corrected, aligning the tracking with the correct road boundary.

**Figure 14:**
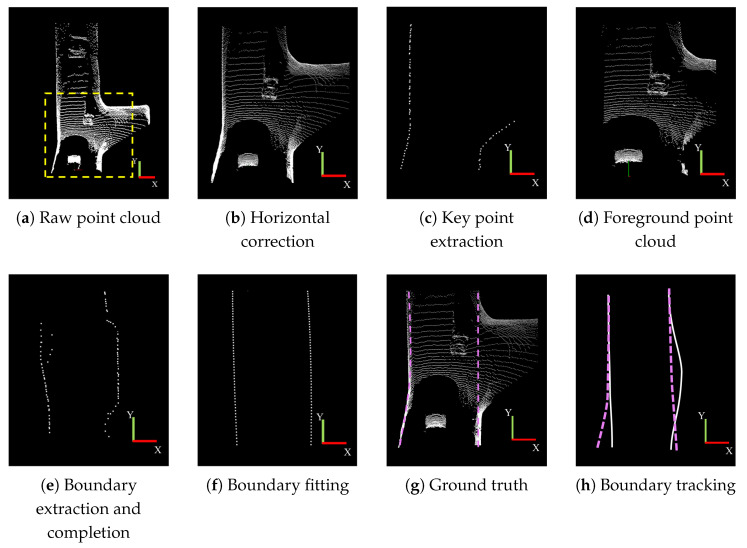
Boundary detection result in the chamber scenario. The true values of boundary fitting are represented in pink, while Boundary fitting is shown in white.

The experimental results for the shunting chamber scenario are illustrated in Figure 14. Figure 14a shows the original point cloud, while Figure 14b presents the data after horizontal correction. In the shunting chamber scenario, the presence of enclosed rooms alongside the tunnel causes interruptions in the extracted key boundary points, as shown in Figure 14c. Consequently, boundary completion operations were also required for the shunting chamber scenario. The process of adaptive boundary completion is depicted in Figure 14d,e. After adaptive completion, the boundary points on both sides become continuous, with the completed results shown in Figure 14f. Figure 14g,h illustrate the boundary fitting results and the manually annotated ground truth, respectively. The fitted boundaries after completion align closely with the ground truth, demonstrating the effectiveness of the boundary completion and fitting processes in this complex environment. Figure 14h presents the boundary tracking results, demonstrating a strong alignment between the tracked boundary and the ground truth.

**Figure 15:**
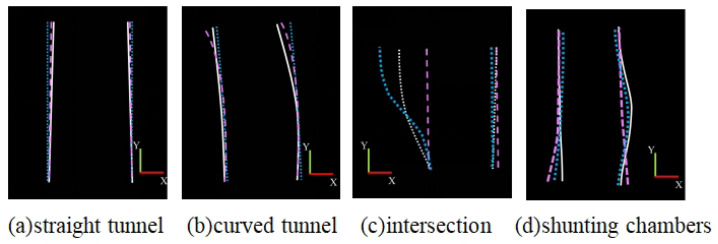
Comparison of different types of roads detected by different methods. From left to right, they are on the straight tunnel, curved tunnel, intersection, and shunting chambers.

Figure 15 shows a comparison between the ground truth and calculated values under four scenarios. The evaluation results of the boundary fitting compared to manually annotated ground truth boundaries for the four scenarios are summarized in Table 2. The straight road scenario achieved the highest precision at 97.5% and required the shortest processing time of 29 ms. The intersection scenario exhibited the lowest precision at 85.0% and took the longest to process, requiring 45 ms. The curved tunnel and shunting chamber scenarios achieved precisions of 93.2% and 88.3%, with processing times of 30 ms and 45 ms, respectively.

The three formulas are presented as follows, with their syntax checked and confirmed to be correct:

(19) $$MSE=\frac{1}{n}\sum_{i=1}^{n}{\left(y_{i}-{\hat{y}}_{i}\right)}^{2}$$

(20) $$\mathrm{Recall}=\frac{TP}{TP+FN}$$

(21) $$F1=2\times \frac{\mathrm{Precision}\times \mathrm{Recall}}{\mathrm{Precision}+\mathrm{Recall}}$$

Here are the explanations of names and symbols in the formulas: MSE (Mean Squared Error) is a commonly used metric to evaluate the accuracy of a regression model; in the context of this study, it quantifies the average of the squared differences between the actual boundary positions ($y_{i}$) and the predicted boundary positions (${\hat{y}}_{i}$) for *n* sample points, with a smaller MSE value indicating a better fit between the predicted and actual boundaries. $y_{i}$ represents the actual measured value of the boundary position for the *i*-th sample point, while ${\hat{y}}_{i}$ denotes the predicted value of the boundary position for the *i*-th sample point obtained using the proposed method, and *n* is the total number of sample points used for error calculation. Recall (Sensitivity or True Positive Rate) is a metric used to evaluate the performance of a classification model in identifying positive instances; in this study, it reflects the proportion of actual boundary points that are correctly detected as boundary points, where TP (True Positive) is the number of actual boundary points that are correctly identified as boundary points by the method, and FN (False Negative) is the number of actual boundary points that are incorrectly identified as non-boundary points by the method. F1 score is a comprehensive metric that combines precision and recall to evaluate the overall performance of a classification model, balancing the trade-off between them and providing a single value to assess the model’s effectiveness, with a higher F1 score indicating better overall performance in detecting boundary points. Precision (Positive Predictive Value) is another important metric for classification models, representing the proportion of predicted boundary points that are actually boundary points. The results in four scenarios are summarized in Table 3.

In summary, the method proposed has demonstrated outstanding performance across various complex scenarios. In the straight and curved tunnel scenarios, the results show that the proposed method can accurately fit and effectively track tunnel boundaries when no interruptions are present. In the intersection scenario, the increased complexity and diversity of the point cloud data introduce challenges, causing some interference with the left boundary. However, the overall results still exhibit high accuracy. In the shunting chamber scenario, despite boundary interruptions, the proposed method achieves relatively accurate boundary fitting through adaptive boundary completion operations. These experimental results highlight the robustness and high feasibility of the method for underground tunnel boundary detection.

### 4.4. Comparative Experiments and Analysis

The straight tunnels, curved tunnels, intersections, and chamber scenes in underground auxiliary transportation roads are similar to the straight roads, curved roads, and obstacle-blocked roads described in Reference [23]. To further evaluate the effectiveness of the proposed road boundary detection method for underground coal mines, a comparison was conducted with the curb detection method outlined in Reference [23]. The comparative results are detailed in Table 4.

From Table 4, the proposed method exhibits superior performance over comparative approaches across all four operational scenarios, with distinct advantages in curved tunnels, intersections, and shunting chambers. In curved tunnel environments, comparative methods rely predominantly on spatial features such as point cloud height differentials for boundary extraction. However, the inherent surface irregularities and steep gradients in underground settings induce frequent fluctuations in ground point cloud elevations, leading to heightened false detection rates when relying solely on height-based cues. In contrast, the proposed method incorporates domain-specific characteristics of underground tunnels and dynamic road surface variations affecting boundary extraction. Through continuous point cloud updating, it maintains the ground point cloud in parallel alignment with the LiDAR coordinate system’s horizontal plane. Moreover, the algorithm employs a grid-based projection strategy to map 3D point clouds onto a 2D grid map, effectively mitigating the impact of height variations caused by surface unevenness and enhancing boundary extraction robustness.

When addressing boundary discontinuities in intersections and shunting chambers, the proposed method achieves more accurate tunnel boundary fitting via adaptive boundary completion. Notably, the average processing time per frame of point cloud data is approximately 35 ms, representing a 10.3% reduction compared to the comparative method with 39 ms, thereby demonstrating competitive real-time performance.

## 5. Conclusions

This study proposes a method for detecting bilateral tunnel boundaries in underground environments, tailored to address the unique challenges of harsh subsurface conditions. In the preprocessing module, a real-time point cloud calibration mechanism—integrating horizontal correction and dynamic fine-tuning—achieves horizontal alignment of ground point clouds, effectively mitigating boundary misdetection caused by steep slopes and uneven surfaces. Subsequently, a grid-based approach, combined with a dedicated boundary extraction module, enables robust extraction of boundary point clouds. To tackle the issue of boundary discontinuities in intersections and shunting chambers, an adaptive boundary completion method is introduced, ensuring the integrity of boundary features in complex scenarios. The method designs a continuous boundary fitting mechanism, deeply integrating temporal context information to maintain long-term consistency of boundary detection results, ensuring sustained stability under dynamic operating conditions. This mechanism breaks free from the reliance of traditional methods on single-moment data; through dynamic correlation and trend prediction of historical boundary features, it effectively resists the impact of instantaneous noise interference and local feature loss, significantly enhancing the continuity of bilateral boundaries in complex scenarios.

Experimental results validate that the proposed method delivers accurate and reliable bilateral boundary detection across diverse unstructured underground tunnel scenarios—including straight sections, curves, intersections, and shunting chambers—while maintaining excellent real-time performance, meeting the safety requirements of underground autonomous driving. Future research will focus on enhancing the method’s adaptability to extreme environments, such as highly fragmented tunnels or sudden structural changes, exploring synergies with lightweight deep learning techniques to further optimize detection precision and processing efficiency, and validating its performance in larger-scale long-term mining operations. Additionally, we will investigate the potential integration of supplementary sensor technologies (e.g., inertial measurement units for improved pose estimation) and explore compatibility with broader autonomous driving systems to enhance scalability. These efforts aim to provide more robust technical support for underground autonomous transportation systems.

## Figures and Tables

**Table 1:** Pass-through filter parameters.

Filter Field	Filter Range	Inverted
$Y^{\prime \prime }$-axis	(0, 20)	No
$Z^{\prime \prime }$-axis	(−2, 2)	No

**Table 2:** Experimental platform description.

Category	Details
Operating System	Ubuntu 20.04 LTS
CPU	8-core Arm®Cortex®-A78AE v8.2
	64-bit CPU 2MB L2 + 4MB L3
GPU	NVIDIA (Santa Clara, CA, USA) Ampere architecture with
	1792 NVIDIA CUDA®cores and 56 tensor cores
Memory	32GB 256-bit LPDDR5 204.8 GB/s
CUDA Version	CUDA 11.3
cuDNN Version	cuDNN 8.6
Python Version	Python 3.9
Development Environment	PyCharm 2023.3
Other Libraries	NumPy 1.26.4, OpenCV 3.4.9.31, PyTorch 2.3.1

**Table 3:** Metrics for the method in four typical scenarios: precision, recall, F1-score, Mean Squared Error (MSE), time (in milliseconds).

Scenarios	Precision	Recall	F1-Score	MSE	Time (ms)
Straight road	97.5%	96.9%	97.2%	0.015	29
Curved road	93.2%	91.0%	92.1%	0.062	30
Intersection	85.0%	81.8%	83.0%	0.18	45
Shunting chambers	88.3%	85.11%	86.7%	0.12	45

**Table 4:** Comparison of detection performance across different methods: precision metrics in straight tunnels, curved tunnels, intersections, and shunting chambers, with average processing time per frame (ms).

Methods	Precision				Average Time per Frame (ms)
	**Straight Tunnel**	**Curved Tunnel**	**Intersection**	**Shunting Chambers**	
Proposed method	97.5%	93.2%	85.0%	88.3%	35 ms
Comparison method	92.0%	83.6%	72.3%	72.8%	39 ms

## Data Availability

The full dataset used in this study cannot be publicly shared. However, to facilitate the understanding and verification of the proposed method, some sample test data can be obtained by contacting the first author (Miao Yu). Researchers interested in accessing the sample data are kindly requested to reach out via the corresponding contact information provided in the manuscript.
